# Planetary healthy publics after COVID-19

**DOI:** 10.1016/S2542-5196(21)00050-4

**Published:** 2021-04-08

**Authors:** Stephen Hinchliffe, Lenore Manderson, Martin Moore

**Affiliations:** a College of Life and Environmental Sciences, University of Exeter, Exeter, UK; b College of Humanities, University of Exeter, Exeter, UK; c Wellcome Centre for Cultures and Environments of Health, University of Exeter, Exeter, UK; d School of Public Health, University of the Witwatersrand, Johannesburg, South Africa

## Abstract

COVID-19 is a sign of a global malaise. The pandemic is an outcome of what we term a planetary dysbiosis, for which underlining drivers include inequality and the exploitation and extraction of human and non-human labours. The implication is that the usual fixes to outbreaks of infectious diseases (ie, surveillance, pharmaceutical measures, and non-pharmaceutical measures) will be insufficient without a thorough reappraisal of and investment in planetary health. Given the heterogeneity and diversity of environments and populations, we envisage these actions as a matter for the generation of new kinds of public, requiring widespread and multiple forms of engagement to generate lasting solutions. We use and extend the concept of healthy publics to suggest a movement that can start to reclaim planetary health as a collective and ongoing issue.

## Introduction

The COVID-19 pandemic, a crisis that has long been imminent, was quickly taken up as a portent of change, a “dress rehearsal”^1^ for recurrent and planetary scale challenges to the lifestyles and norms that are associated with late modernity.^1^ But what hope can a pandemic generate, and what good might we expect in reconfiguring or recomposing planetary public health in its aftermath? We write this Personal View in the belief that healthy publics, a term that is used to describe “dynamic collectives of people, ideas and environments that can enable health and well-being”,^2^ can inform this recomposition. The pandemic has helped to foreground social and ecological relations and has provided new impetus to the task of identifying relationships that are crucial to life and health.^3^, ^4^ Here, we set out some principles to make sure that these matters (ie, socioecological relations and the life-support systems on which we all depend) retain saliency and public concern. We start by tracing the numerous problems with a business-as-usual approach, which is so often combined with versions of biomedical, technological, or behavioural fixes to planetary problems. We then outline the terrain of a planetary health approach before insisting that, beyond a simple grouping together of human, non-human, and environmental concerns, there is a need to consider how planetary healthy publics can be assembled and sustained. How, in other words, can a more active reconstitution of health be generated in the wake of the COVID-19 pandemic?

## Building back differently: the limits to the biomedical, technical, and behavioural sublime

Diseases tend to usher in new eras, paving the way for new forms and waves of public health programmes and even new disciplinary practices. John Snow's evidence of the link between cholera and contaminated drinking water from the Broad Street pump, for example, provided the evidential and material bases for responses to recurrent epidemics, the development of epidemiology, and reforms of public health infrastructure on a large scale in mid-19th century England.^5^, ^6^ Robert Koch's and Louis Pasteur's articulations of germ theory, although initially contested, consolidated practices of disease surveillance, case isolation, disinfection, and hygiene education to strengthen the role of the state in containing disease in imperial nations and their outposts during the late 19th and early 20th centuries.^7^, ^8^, ^9^, ^10^ The subsequent development of sulphonamides and later antibiotics provided new confidence in the so-called metropolitan conquest of microbes in the 1930s.^11^, ^12^ Thereafter, the growing effectiveness of therapeutics and prevention, including vaccination and eradication campaigns during the second half of the 20th century, created a modern sensibility that any disease was preventable.^11^, ^13^, ^14^ This confidence in biomedicine and its technologies has continued into the 21st century, and it has been applied to animal health practices, where enclosed, high-throughput, and tightly monitored livestock production is often presented as a fix for epizootics and zoonoses.^15^, ^16^

This modern sensibility is likely to persist even in the aftermath of this pandemic.^17^ As an example, the lure of the so-called vaccine narrative^13^ established itself within COVID-19 from the outset, concentrating efforts to develop vaccine candidates and collapsing the time to test, trial, and gain regulatory approval for the resulting vaccines. At present, vaccines are rolling out at previously unimaginable speeds, providing people who are inoculated with some protection from severe COVID-19. This incredible achievement is of course to be celebrated, but just as importantly, there is a need to remember that vaccines enter into relationships with microbial selection processes, immune responses, and populations, and they are dependent on the socioeconomic and political dimensions of vaccination programmes. Indeed, upper respiratory mucosal infections involving SARS-CoV-2 are predicted to continue in vaccinated and recovered hosts, and antigenic escape variants will require vaccine adjustments for some time to come.^18^ Successful vaccination programmes depend on attendant infrastructures (ie, cold chains, clinics, materials, labour, and clinical waste management) and many other social conditions. Geopolitics, pharmaceutical markets, structural inequalities, and constraints on health systems continue to influence access and purchase agreements, reduce country capacity for distribution, and undermine vaccine viability and distribution. Social media exposure of people who are advocating for vaccine hesitancy and mistrust of biomedicine and corporate health governance, and the emergence of so-called infodemics (ie, the overabundance of both online and offline information, much of which can be knowingly false) that relate to conspiracy theories, only add to this complex of issues. Involuntary movements of populations, forced migration, and semipermanent refugee camps; civil war; economic, political, and other pressures; absence of civil status; and fragile governments all add to the difficult ends of disease.^19^ Although many programmes for disease control have historically had eradication as a goal,^20^, ^21^ it is worth remembering that few have succeeded.^22^, ^23^ Moreover, the rise of drug-resistant infections fuels growing concern that the intense use of antimicrobial therapeutics, including during the current pandemic, might accelerate a demise in the effectiveness of important antibiotics.^24^ SARS-CoV-2 is simply one of many microbes where sustained social changes are a key component of disease management. Faith in narrowly framed pharmaceutical fixes to planetary health problems is simply misplaced.

**Key messages**

• COVID-19 is a sign, not a cause, of a general malaise.
• Biomedical and technical fixes will be ineffective unless underlying drivers of planetary dysbiosis are confronted.
• Pandemic preparedness should address the inequalities, governance failures, and structurally generated ill health that make the planet more infectable.
• Modernisation of health provision and agriculture to reduce pathogen emergence will fail in the absence of attempts to reduce the levels of extraction and exploitation of people, other living organisms, and environments.
• A planetary healthy public is a term that we use to signal the need for a process of generating new forms of investment in, engagement with, and political deliberation over healthy planetary futures.

Immediate and ongoing COVID-19 responses initially involved—and revived well rehearsed and somewhat ancient—non-pharmaceutical approaches to disease control. The oddly termed social (ie, physical) distancing, enhanced hygiene, travel restrictions, shielding, and isolation speak to a shared understanding of spatial separation to interrupt virus transmission. These injunctions echo earlier hisorical policies of *cordon sanitaire*, effected to control national borders and in the context of local and colonial public health.^25^ Yet, the ability to generate and sustain distance and to shelter in place (ie, as recommended in US threat management for populations at risk) is unevenly distributed and conditioned by setting, gender, income disparities, working conditions, climate, political trust, access to sanitary infrastructure, and compliance to public health messages. Meanwhile, for non-human populations, the likelihood of diseases emerging or being amplified within spatially sequestered, biosecure, sites of intense production are becoming increasingly apparent.^26^, ^27^ As for pharmaceutical interventions, non-pharmaceutical measures are only as good as social and environmental conditions allow.

Another category of response to epidemics and pandemics is the rise of smart systems, mobilising early warning through manipulation of big data for and real-time surveillance of emerging diseases. For some, the future of public health is likely to become increasingly digital,^28^ and blockchain and other developments that are related to artificial intelligence offer potential to trace and monitor the health of systems for food production.^29^ Early warning systems have for some time been seen as enabling rapid deployment of pharmaceutical and non-pharmaceutical measures to mitigate the effects and extent of epidemics.^30^ However, species, microbiological, and symptomatic diversities; the structural limitations to pharmaceutical fixes; security concerns surrounding dual use research; doubts regarding uses of public health data; and the economic margins that affect most of the world's food production make the application of smart systems a partial solution at best. The introduction of smartphone apps to track contacts for COVID-19 have faltered even in smart cities, such as Singapore; barely got off the ground in some countries, including Australia and the UK; and faded out as a technology of containment as rapidly as they were introduced. High-technology surveillance systems for farming will do nothing about the levels of extraction of value and exploitation of labour of human and animal bodies that are at the root of a system that is permanently close to failure. A key question concerning the promise of digital technology as a tool for public health, or even as some version of the technological sublime (ie, the awe and wonder that is sometimes attached to a technological promise or achievement), is how frequently it emerges and is then dashed by the practical and ethical difficulties of its realisation.^31^ The crucial question is therefore not how digital technology might deliver public health, but how a healthy public might arise from new forms of communication and knowledge generation.

If the focus of response to infectious disease has tended to be on technically mediated sanitation and separation, the slow epidemics of chronic and non-communicable diseases have largely concentrated public health interventions around risk factors and the increased responsibilities of individuals and culturally defined groups. These frameworks of risk and lifestyle—concepts and strategies that spread from the USA, the UK, and colonial research centres in the postwar period^32^, ^33^, ^34^, ^35^—have long obscured the complex relational and structural factors underpinning rising rates of conditions such as cancer and cardiometabolic diseases^36^, ^37^ and the production of syndemics.^38^, ^39^, ^40^, ^41^ Further, the prevalence of these conditions shows how national economies have long been integrated into imperial and global economies.^42^ Within such systems, low-income countries have been pushed to sell food to high-income countries and to import less nutritious alternatives than were exported.^43^, ^44^, ^45^ Poor communities within and across nation states have thus been forced into food dependency and narrow food choices, with inequitable or non-existent access to the environments^46^ or temporal and material resources^47^ that are required for health. They have, in other words, no choice of lifestyle.^41^ Low income, high food costs, scarcity of access to cooking facilities or to non-toxic foods, and living in crowded conditions have all meant that self-care and disease-preventive behaviours through self-governance are primarily a privilege of a wealthy few. Disease risk and prevalence still track the faulted and fractured terrains of race, gender, class, and other conditions that are shaped by uneven distributions of power.

## On planetary health

Within a planetary health approach, which “requires judicious attention to the human systems” that shape environments and condition human flourishing,^48^ SARS-CoV-2 and the resultant COVID-19 are signs and not causes of a global malaise. Pathogens, such as SARS-CoV-2, are the result of a planetary dysbiosis and reflect much more than the effects of contamination or the unencumbered viral traffic (ie, the transmission and spread of viruses from the points of origin, often through transport, trade, and travel) that shaped much of the 20th century's discourse on emerging infectious diseases.^49^ In contrast to a focus on contact and transmission across smooth surfaces (ie, the idealised mathematical surfaces of epidemiological transmission models), a planetary approach suggests a more complex terrain of a disease.^50^, ^51^, ^52^ A terrain, for the philosopher of medicine, Georges Canguilhem, is a bodily predisposition that is more or less receptive to, and so defines the severity and effect of, a disease. Extending Canguilhem's definition, terrain can also usefully underline the planetary aspect of this condition of so-called infectability (ie, the extent to which an organism is vulnerable to becoming infected).^15^ This focus includes linking the emergence of SARS-CoV-2 and similar novel viruses to habitat destruction, illegal trade in wild animals, climate instabilities, and changing intensities of the relationships between humans and other animals.^53^ The focus also allows consideration of how historical and socioeconomic conditions shape predispositions of infectability. As the COVID-19 pandemic has shown, these conditions include structural inequalities that cause poverty and racial discrimination and determine living conditions, and globalisation and market-based inequities that shape relationships to animals and landscape. They include governance issues, such as the ways that local and national states are able or enabled to respond to emergencies and to the long-term processes that denigrate health and environment. The list of proximal and distal causes to any pandemic can be exhaustive and exhausting, and can make systems, syndemic, and One Health thinking seem little more than a grouping of many risk factors. Nevertheless, this nexus of causes underlines that planetary health futures cannot be fixed by pharmaceutical means alone or by imposing and policing boundaries between populations or species, even if these fixes were technical, economic, logistic, and administrative possibilities. Disease, health, and their terrains are much broader than this contamination view tends to imply.

The current pandemic emphasises the extent of the challenges that will intensify in the coming decades. The recrudescence of previous infectious diseases will continue as a result of inadequate infrastructure that will struggle to cope with population distributions and mobilities, changes to the environment that are associated with human activities, and climate change. The rapid spread of dengue virus globally in the past half century is one such example; mobile infected hosts, vectors, and larvae and landscape changes that are associated with periurbanisation and edgeland development, which favour vector and parasite lifecycles, have added to the social hyperendemicity of the disease.^54^, ^55^ This altered terrain for disease might be amplified by climatic and oceanic cycles and rapid changes to climate. In southern Africa, warm El Niño–Southern Oscillations are associated with the increased incidence of malaria, cholera, and Rift Valley fever virus; La Niña is associated with dengue virus, chikungunya, Zika virus, and yellow fever. Add to these shifts the, now wildly underestimated, increased number of people who are living in extreme poverty, from 6·2 million people in 2016 to 18·7 million people in 2030, and we have a disastrous mix in terms of pandemic potential (ie, emergence of pathogens and highly infectable human populations).^56^ For example, decreased or disrupted supplies of potable and other water will affect hygiene and sanitation, increasing the risk of water-washed diseases (eg, dysentery, scabies, trachoma, conjunctivitis, skin infections, and ulcers) in poorly resourced nations, settings, and households. Depleted water supply will also affect commercially produced food and subsistence production, both urban and rural, affecting local and national food security, food pricing, nutrition, and health. Compromised immune responses, which are themselves associated with multiple health challenges and acute and chronic impoverishment, add to this challenge.

COVID-19 has made it clear that a so-called modern sensibility or eradication mindset that is bolstered following infectious disease events, and the tendency to divide and individualise health responsibilities, is insufficient. It has also become clear that radical extension and intensification of distancing, both in terms of human–non-human contact and within social worlds, is socially, economically, and environmentally unsustainable. A public that divides and sequesters humans, excludes anything other than what is narrowly thought to be human (or human made), or even zones a planet into spaces for humans and spaces for wildlife severs the relationships that make life possible.^57^ Calls, for example, to reinforce a fortress nature approach (ie, a form of conservation involving protected areas that are isolated from people) ignore or discount the people who live within biodiversity hotspots, who are often intrinsic to making those landscapes biodiverse.^58^ This spatial fix also leads to renewed xenophobia, the withdrawal of humanitarian efforts, and, similar to antecedent efforts to construct such enclaves in colonial settings,^59^ bubbles and exclusions, which would most likely be built on conceptualisations of others (ie, people who are defined by powerful groups as existing outside of conventional social orders) as dangerous sources of disease.^8^, ^60^ The consequence is to repeat and rematerialise the racialised, classed, gendered, and other exclusions to humanity that were central to imperial, extractive regimes^61^, ^62^ and perpetuate the modernist fiction that life was possible without the metabolic and other relationships that rely on diverse living microorganisms and macroorganisms and their terrains.

These approaches all share an ontological view of disease, as matter to be driven out through spatial separation. They involve re-establishing a norm through battle with an identified pathogen.^63^ An alternative would be to focus less on disease as an existential threat to planetary health, and more on planetary health as a dynamic process and an adaptation to disease. This approach is similar to considering health and disease “not so much as qualitatively opposed, or as forces joined in battle”,^50^ but as opportunities for establishing new norms or counter norms. What these counter norms will or can be is, we would suggest, a matter for planetary healthy publics.

## Healthy planetary publics

A convention in public health has been to talk about the public as a singular target (ie, of health interventions) or as a population (ie, a calculable entity of potential or existing cases).^2^, ^64^ Public health campaigns to keep safe, stay home, and stay alert, or public health epidemiology referring to basic reproduction numbers and herd immunity, instantiate these approaches. If, or when, non-human species or environments enter into this logic, they do so in a similar manner, to warn of toxicity, to encourage biosecurity in livestock, or to model zoonotic transmissions. However, publics can also be understood as groupings of people, their non-human counterparts, and ecological and social relations.^65^, ^66^ These assorted and collective heterogeneous publics are, sociologist Noortje Marres has argued, “sparked into life” by an issue.^67^ Following this formulation, COVID-19 can be said to have momentarily encouraged a planetary healthy public. The pandemic has mobilised pre-existing debates on the role of wildlife and habitat denigration in generating viral spillovers into the human population. But COVID-19's hotspots (and sparks) have not ended there. The unequal and often tragic clustering of cases within particular sites and demographic groups has produced new conflagrations or disease hotspots, including in care homes; meat-packing plants; indigenous communities; and nation states where populist forms of government have increased indecision, intensified the outsourcing of public health provision, and had difficulties in generating meaningful compliance with measures for disease response. The continued relevance of race, poverty, identity, and violent racism to unequal health outcomes has compounded these social and ecological complexes of disease interactions.

So how might a healthy planetary public be sustained following a devastating pandemic? The source for this way of thinking about publics, as generative of new norms, derives from the pragmatist philosophy of John Dewey,^68^ in which publics emerge and then struggle to sustain themselves. The “new public which is generated remains long inchoate, unorganized, because it cannot use inherited political agencies. The latter, if elaborate and well institutionalized, obstruct the organization of the new public… To form itself the public has to break existing political forms... so often effected only by revolution”.^68^ It is clear that a new kind of public that takes the demarcations of human, non-human, and their inter-relationships as matters of importance and open contestation is necessary for planetary health. Our final intention is to outline some of the signs of its inception and note current obstructions that will need, in Dewey's sense, to be broken should planetary healthy publics be realisable.

First, the present pandemic draws attention to the need for massive new investment in public health, its institutions, settings, and, crucially, workers. The differential ramifications of the disease have emphasised that public health and healthy publics are tenuous while mass global inequality is sustained. Revaluing health-related activities requires a shift from neoliberalism and self-care^69^ to attend again to the systems and services that help to sustain public health. In some countries, governments, and to an extent the private sector, have partially and tentatively reversed policies of neoliberalism to ensure survival through the pandemic. In the early months of the pandemic, in Australia, job-seeker and job-keeper payments were introduced at levels that acknowledged the inadequacy of unemployment benefits and pensions before COVID-19; banks restructured mortgages and reviewed repayments on loans; child care was provided free to essential workers; and so on. In South Africa, quickly after the initiation of lockdown, small grants were provided in acknowledgment that people without resources would be most seriously affected by economic contraction and social constraint. In these contexts, an emergent public has begun to question whether a government can, and should, simply revert back to austerity and other monetarist policies after the pandemic, given the probable continued massive unemployment and poverty that are directly associated with the measures that were put in place to manage viral spread and with the structural inequalities that pre-date the pandemic. The pragmatic responses to COVID-19 to ensure social survival might not lead to embracing a strong programme of social welfare, but such fiscal interventions have opened up a debate about the roles and legitimacy of the state, their relation to the private sector, and the priorities and obligations of international aid.^70^

Second, a planetary healthy public requires a recalibration of focus and attention on entities other than humans and their inter-relations. Renewed attention to global heating, disruption in ecospheres, and loss of biodiversity has led to more frequent statements at national and multilateral levels concerning the urgency of human interventions in these domains through changes in how we live on the planet. A radical shift in relations to non-human animals and environments will be necessary if, for example, we are to reduce the emergence of, and the terrain that is amenable to, new and emerging diseases. This approach is public health as One Health, requiring changes to how and in what ways people cohabit with domestic and wild animals and plants. These changes will require considerable reappraisal and perhaps international, Marshall-like planning (ie, a programme to provide economic rescue on a large scale) to reconfigure food production and accessibility. Large corporate and intensive food producers, profiteering from highly transmissible diseases, such as African swine fever virus,^71^ are not the answer to a pandemic planet; they are part of the problem.^15^, ^26^, ^72^

Third, as existing national political forms have been forced into temporary changes, a potential severing of established supranational forms of cooperation and coordination can be witnessed. How global bodies might continue to provide leadership, or work with other institutions, is evolving, not least at the 73rd World Health Assembly, which was held virtually for the first time in its history in May, 2020, and focused only on one disease for the first time ever (and not more broadly on health issues as in its constitution). At the same time, WHO has not been subject to the same amount of criticism as was the case in relation to the 2013–16 Ebola virus outbreak. The Zika virus pandemic, which was first recognised in 2013, was perhaps a rehearsal for managing the spread of SARS-CoV-2, but all of these viruses, and especially SARS-CoV-2, emphasise the limits to action and the vulnerability of international agencies when their operating funds are subject to national policies and political whim. In this context, the equivocation and threat of withdrawal of support from nation states to WHO reflect the fragility of international conventions and agreements, including those central to a global response to planetary health. The challenges are sizeable: to speak of suprastate bodies as both accountable and publicly oriented is difficult. Their displacement by, or partnerships with, philanthropic capital make democratic accountability even less apparent and can further skew the aims and methods of public health towards the biomedical and the technological sublime.^73^, ^74^

Fourth, rethinking and generating healthy publics will require infrastructural change on a large scale, not least an end to neoliberal political economy and a renewed collectivisation of health and financial risk. Healthy publics will require cultural transformations in understandings and practices of health, similar to the rise of biomedicine in the 19th and 20th centuries. The UK experiment in the de-facto privatisation of health care and pandemic response has done little to endear people to a form of health care that is poorly adapted to local conditions, devoid of necessary expertise or capacity, and seemingly prone to levels of cronyism and corruption. The question in a world after COVID-19 is not whether change is needed but how to bring it about in an engaged and participatory manner.

## Concluding points for action

Although potentially important in terms of raising issues and particularities relating to health and wellbeing, healthy publics can seem somewhat locally specific. Faced with the prospect of planetary survival and the heterogeneities and cultural specificities of global populations, the notion of planetary healthy publics might appear misplaced. And yet, the current predicament and the imminence of climate and other forms of crisis mean that there is a need to generate new kinds of public conversation and experimentation with living together differently. To conclude, we present a few observations and jumping-off points that COVID-19 events have sparked into life and that, similar to new innovations in vaccine technologies, should be candidates for rapid testing and roll out.

COVID-19 has upturned perceived notions of what it takes to be prepared. Pandemic preparedness plans were introduced from 2003 with SARS-CoV, and revised in 2009 in the context of H1N1 influenza. The country that was allegedly one of the most prepared according to the Global Health Security Index—the USA—continues to lead, at unimaginable rates and numbers, the toll in new cases and mortality from COVID-19. A first step to generate planetary healthy publics is to support open discussion of what preparedness might entail and how the provision of health care within communities, and between nations, needs to be addressed.

We would start that conversation by suggesting that preparedness incorporates a measure of socioeconomic deprivation and inequality. Inequality as a key driver of disease should dominate any epidemiological analysis of the pandemic. The issue is gaining new momentum through organised cultural movements, such as Black Lives Matter, and will most likely steer political change worldwide. Following the economics of Picketty and Goldhammer,^75^ it is time to invest in health equity for economic, planetary health and wellbeing, and ethical reasons.

Human exceptionalism is a key driver of disease emergence and transmission. We need a radical recalibration of what it means to be human and so should develop new resources for rethinking human health in terms of planetary health. Planetary health, like One Health, is rarely a positive sum game.^76^ There will be trade-offs and undoubtedly conflicts as we continue to explore questions of how to live together. This inherently open and contested aspect means that planetary health is an interdisciplinary, transdisciplinary, and political process that will involve difficult choices, confrontations, and redistributions.

Finally, publics are, by their nature, spaces for disagreement and agreement. A healthy public is not advocacy of a popular will or a suggestion that planetary healthy publics can be constructed in any desired way; far from it. Forming a planetary healthy public will involve political struggle to contest those versions of the future that are offered as somehow inevitable or beyond politics.^77^ The politics of planetary public health demand a shift away from the biomedical, technological, and behavioural fixes and the economic imperatives of market capitalism, towards forms of governance and organisation where the health of the planet is assessed openly in terms of its equitability and sustainability and in its ability, as philosopher Isabelle Stengers suggested, to avoid the barbarism that is so often presented as destiny.^77^

## Declaration of interests

We declare no competing interests.
