# Correction to *Lancet Planet Health* 2021; 5: e230–36

**DOI:** 10.1016/S2542-5196(21)00146-7

**Published:** 2021-06-10

*Hinchliffe S, Manderson L, Moore M. Planetary healthy publics after COVID-19.* Lancet Planet Health *2021; **5:** e230–36—*In this Personal View, the Acknowledgments section should have read “The Wellcome Trust funded the Centre for Cultures and Environments of Health (grant 203109/Z/16/Z), allowing SH and MM time to work on the Review. The funder of the study had no role in study design, data collection, data analysis, data interpretation, or writing of the report.” This correction has been made as of June 10, 2021.

